# Unveiling Genetic Loci for Root Morphology and Salt Response at Rice Seedling Stage via Genome-Wide Association Studies

**DOI:** 10.3390/life15101595

**Published:** 2025-10-13

**Authors:** Zifan Xue, De Hao, Zheyu Lu, Jie Yang, Ziteng Geng, Chengsheng Meng, Yanru Cui

**Affiliations:** College of Agronomy, Hebei Agricultural University, Baoding 071000, China; 13021867098@163.com (Z.X.); 18395698106@163.com (D.H.); 18279856187@163.com (Z.L.); yangjie0417163@163.com (J.Y.); 15632882220@163.com (Z.G.)

**Keywords:** *Oryza sativa*, salt tolerance, root morphological phenotypes, GWAS, QTNs

## Abstract

Rice (*Oryza sativa* L.) is a salt-sensitive crop, where even moderate soil salinity (electrical conductivity ≥ 3.5 dS/m) can cause significant yield reduction. During the seedling stage, the underdeveloped root system has limited capacity for salt uptake and translocation, making root system architecture (RSA) a crucial trait for enhancing salinity tolerance. In this study, we used 165 individuals from the 3K Rice Genome Project to comprehensively measure multidimensional root morphological traits at the early seedling stage under salt stress, thereby overcoming the limitations of conventional methods that mainly rely on root length and biomass. We identified 78 quantitative trait nucleotides (QTNs) associated with eight root morphological traits through genome-wide association studies (GWAS) of 3VmrMLM. Among these, 12 QTNs co-localized within genomic regions of previously cloned salt tolerance-related genes. Additionally, six salt-tolerant lines were selected based on significantly increased root volume (RV) and surface area (SA), suggesting that their adaptive mechanism under salinity involves optimized spatial root distribution rather than radial thickening. Our findings show that high-resolution root scanning-based phenotyping provides a reliable platform for screening and breeding salt-tolerant rice varieties, offering valuable indicators for assessing seedling-stage salt tolerance.

## 1. Introduction

Rice (*Oryza sativa* L.) is a staple crop vital to global food security, feeding more than half of the world’s population [1,2,3,4,5]. In China, rice production exceeded 200 million tons in 2023, accounting for over 40% of the national total grain output. However, soil salinity poses a severe threat to rice productivity worldwide. Approximately 20% of irrigated farmland is already affected by salinity, with this area expanding by 1–2% annually due to climate change, sea-level rise, and unsustainable agricultural practices [5,6,7]. Globally, saline-affected soils cover about 950 million hectares, nearly 100 million of which are in China, including 15 million hectares potentially suitable for crop production, representing roughly 10% of the country’s arable land [8]. Cultivating salt-tolerant rice varieties is therefore crucial for enhancing food security and enabling agricultural use of marginal lands [9,10].

Rice is highly sensitive to salt stress [5,6]. An electrical conductivity (EC) as low as 3.5 dS/m can lead to a 10% yield reduction, while EC levels of 7.2 dS/m may cause losses of up to 50% [11]. The seedling stage is particularly vulnerable: roots are underdeveloped and less capable of ion regulation and transport, making early salt tolerance indicative of whole-plant resistance [12]. Since salt tolerance is a polygenic trait, uncovering its genetic basis is essential for breeding resilient varieties [13,14].

GWAS has been a powerful tool for dissecting complex traits [15,16]. Recent research has identified multiple QTNs and genes related to salt tolerance at various rice growth stages. For example, some studies have highlighted the role of root-specific traits and ion transporters such as *OsHKT1;5*, *OsMKK10.2*, and *OsWRKY53* in sodium exclusion and homeostasis [17,18]. Yu used 268 rice individuals from around the world to identify the genes encoding transcription factor *OsWRKY53* and mitogen-activated protein kinase *OsMKK10.2*, which mediate root Na flux and Na homeostasis [17]. Le identified 26 QTNs through GWAS using 179 Vietnamese rice landraces and 3 control genotypes (Nipponbare, Azucena, and IR64), which are mainly involved in gene regulation, signal transduction, or hormone signaling [19]. Nayyeripasand used 155 rice varieties from the Genetic Resources Center of the International Rice Research Institute (IRRI) in the Philippines to detect 151 QTNs related to eight salinity-related traits, including shoot length (SL), root length (RL), root dry weight (RDW), root fresh weight (RFW), shoot dry weight (SDW), relative water content (RWC), and total weight (TW) [20]. Nevertheless, most traditional phenotyping approaches focus on simple root traits like length, biomass, and absorption of NA^+^ and K^+^, overlooking multidimensional root system architecture (RSA) traits that may be critical under stress.

The root system is the first organ to encounter soil salinity, directly affecting water uptake, nutrient acquisition, and plant establishment. RSA traits, including root volume, surface area, and distribution, strongly influence salinity adaptation and final yield [21]. Amid growing saline-alkali soil constraints on global rice production, the genetic basis of root morphological responses to salt stress remains unclear, and integration of root-associated QTNs, well-characterized salt-tolerance genes, and breeding resources is underexplored [22]. However, recent high-throughput root phenotyping technologies allow detailed morphological characterization, providing new opportunities to link genetic variation to RSA under stress.

In this study, we evaluated a diverse panel of 165 rice accessions from the 3K Rice Genome Project at the seedling stage under salt stress. Using a ScanMaker i850 root scanner and standardized protocols, we collected high-resolution phenotypic data for multiple RSA traits. Integrating these data with genome-wide markers, we conducted GWAS to identify quantitative trait nucleotides (QTNs) associated with salt tolerance. In light of this, the present study addresses the key research gaps mentioned above. We thus identified salt-stress-related root-trait QTNs, validated their overlap with known salt-tolerance genes, and screened salt-tolerant accessions for breeding. Ultimately, this work aims to provide genetic insights and tools for salt-tolerant rice, facilitating saline-alkali soil use and sustaining global rice production.

## 2. Materials and Methods

### 2.1. Materials

A total of 167 core rice germplasm accessions were selected from the 3K Rice Genome Project, including two control materials: FL478 (a salt-tolerant *indica* rice cultivar) and IR29 (a salt-sensitive *indica* rice cultivar). The 3K Rice Genome Project was a collaborative effort among three institutions—the Chinese Academy of Agricultural Sciences (CAAS), the International Rice Research Institute (IRRI), and BGI-Shenzhen—to sequence the genomes of 3024 accessions and landraces of Asian rice (*Oryza sativa*). The experiments were conducted from October 2024 to April 2025 in the greenhouse of the National Key Laboratory of Crop Improvement and Regulation in North China.

### 2.2. Experimental Procedure

The 167 individuals were subjected to a 48 h treatment in a 50 °C drying oven to break seed dormancy. For each individual, 60 plump and uniformly sized seeds were selected, surface-sterilized by soaking in a 2% sodium hypochlorite (NaClO) solution for 30 min, and then thoroughly rinsed with distilled water. The sterilized seeds were evenly distributed in clean seedling trays, supplemented with an appropriate volume of distilled water, and incubated at a constant temperature of 37 °C for 72 h to promote germination, with daily water replacement. After seed germination, 24 healthy seeds with uniform growth status were selected and transferred to a 96-well planting plate. They were initially cultivated in distilled water for 4–5 days, followed by Yoshida’s nutrient solution [23] (Supplementary Table S1) until the three-leaf-one-heart stage. Subsequently, a single salinity concentration was applied to the seedlings: they were cultured in Yoshida’s rice culture solution with NaCl supplementation, resulting in an electrical conductivity of 3.5 dS/m (28 °C) equivalent to 140 mmol/L NaCl, for a 7–12-day period. After treatment, the solution was replaced with a standard nutrient solution. On the third day of rehydration, the growth performance of each line was observed. During this period, the pH was maintained at 5.5–5.8, and the nutrient solution was replenished every 6 days. The experiment included two biological replicates and one blank control, arranged in a randomized complete block design (RCBD).

### 2.3. Phenotypic Data Collection

#### 2.3.1. Salt Tolerance Level Evaluation

Salt tolerance evaluation was conducted according to the Standard evaluation score of visual salt injury at the seedling stage (Supplementary Table S2) [24]. The salt tolerance scale was classified into five grades: Grade 1 (highly salt-tolerant), Grade 3 (moderately salt-tolerant), Grade 5 (intermediate salt tolerance), Grade 7 (salt-sensitive), and Grade 9 (highly salt-sensitive) [24]. FL478 and IR29 were used as salt-tolerant and salt-sensitive control varieties, respectively. Phenotypic observations were recorded three times daily (morning, noon, and evening), and the mean values of the two treatments were calculated as the final statistical data.

#### 2.3.2. Root Morphological Trait Acquisition

For each cultivar following salt stress treatment, six randomly selected plants were subjected to root system sampling. The collected root samples were preserved in ethanol and stored at −4 °C. Prior to measurement, roots were transferred to a transparent hydroponic tray, immersed in distilled water, and carefully dispersed to avoid overlapping. Root architecture was digitally captured using a ScanMaker i850 plus root scanner (Microtek, Caohejing New and High Technology Development Zone, Shanghai, China) with sequential scanning of all samples. The acquired high-resolution images were saved for subsequent analysis. Quantitative assessment of root morphological parameters was performed using the WinRHIZO root image analysis system (Regent Instruments, Quebec City, QC, Canada). A total of eight root architecture traits were obtained: Root length (RL) represents the arithmetic mean of the total root length in the image; Root diameter (RD)is the arithmetic mean of the diameters of all scanned root segments, reflecting the overall thickness of the root system; Root crossings (RC) refers to the number of self-crossing points identified via image analysis, where roots overlap in the two-dimensional projection—a higher value indicates more branched and spatially complex root systems; Root forks number (RFN) denotes the number of nodes where a root segment bifurcates into two sub-segments, with higher values suggesting greater topological complexity; Project area (PA) is the vertical projection area of the root system on the two-dimensional scanning plane, indicating the horizontal distribution range of the roots; Root volume (RV) is calculated based on root diameter and length data, representing the total soil space occupied by the roots; Surface area (SA) is derived from scanned root diameters, with larger values reflecting a greater interface for root–soil contact; Root tips number (RTN) indicates the number of unbranched root apices, where higher counts suggest active root growth and exploratory capacity.

### 2.4. Data Analysis

The data were collated and statistically analyzed using Microsoft Excel 2010. Salt tolerance-associated genetic loci in rice seedlings were identified using R 4.4.1 software. A single-locus GWAS was performed at the SNP level employing the 3VmrMLM method. The 3VmrMLM method, developed by Zhang Yuanming from Huazhong Agricultural University, is a compressed variance component mixed model method [25]. It is capable of estimating additive and dominant effects, along with their environmental and epistatic interaction effects, while fully controlling for all possible polygenic backgrounds. We employed this method to detect main additive effects through a two-step procedure: first, a single-locus model identified potential SNPs associated with the target traits using a threshold of 1 × 10^−5^; second, a multi-locus model, which performed only one statistical test incorporating all the potential SNPs from the first step, was used to identify the final QTNs with a threshold of 0.05.

## 3. Results

### 3.1. Descriptive Statistical Results

A total of 6 salt-tolerant individuals were screened among the 165 rice materials. Descriptive statistical analysis was carried out on eight root scanning traits of the rice root system. The coefficients of variation for the eight traits ranged from 10.34% to 46.70%. Except for the RD, the coefficients of variation for the other traits were above 11%. The variation range of coefficients of kurtosis and skewness for eight traits was 0.06–1.51 and 0.49–0.98, respectively, indicating that the 8 traits basically follow a normal distribution. Three of the eight traits (PA, SA, and RTN) had relatively concentrated phenotypic data, with the following ranges: 3.34–11.64 cm^2^, 10.50–36.56 cm^2^, and 346.50–2792.00. The other five traits—namely, RD, RC, RFN, RL, and RV—exhibited relatively scattered phenotypic data. The phenotypic values ranged from 0.21 mm to 0.41 mm, 30.50 to 389.50, 238.50 to 1819.50, 109.54 cm to 439.94 cm, and 0.07 cm^3^ to 0.29 cm^3^, respectively (Supplementary Figure S1, Table 1).

### 3.2. Correlation Analysis Results

**Figure 1:**
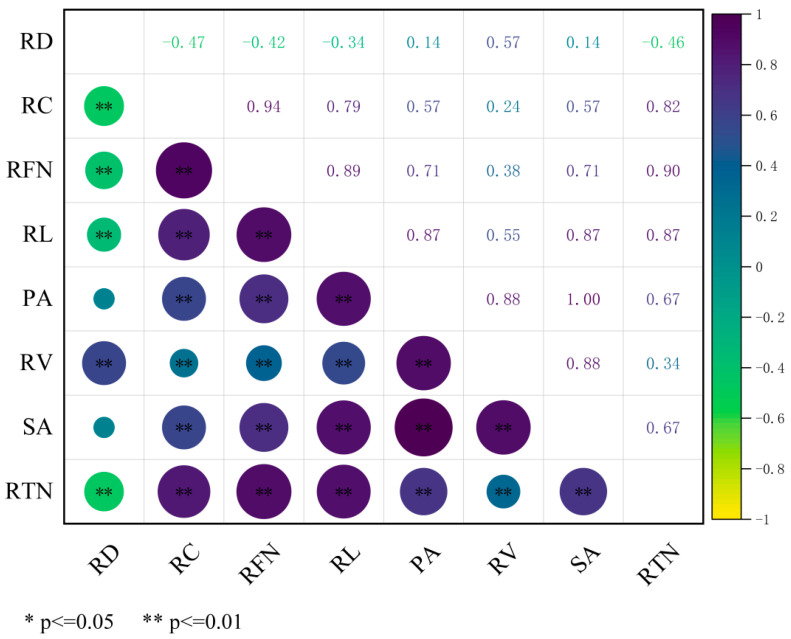
Results of correlation analysis on root characteristic traits. Note: RD, Root diameter; RC, Root crossings; RFN, Root forks number; RL, Root length; PA, Project area; RV, Root volume; SA, Surface area; RTN, Root tips number.

Correlation analysis revealed a complex pattern of associations among the root morphological traits (Figure 1). RL was significantly negatively correlated with RD (*r* = −0.35) but exhibited significant positive correlations with the other six traits: PA (*r* = 0.88), SA (*r* = 0.88), RTN (*r* = 0.87), RFN (*r* = 0.89), RC (*r* = 0.79), and RV (*r* = 0.57). A strong positive correlation was found between PA and SA (*r* = 1.00). PA was also positively correlated with RV (*r* = 0.89), RFN (*r* = 0.72), RTN (*r* = 0.68), and RC (*r* = 0.58). Similarly, SA showed identical correlation coefficients with RV, RFN, RTN, and RC as PA. The trait of AD demonstrated a distinct correlational pattern: it was positively correlated with RV (*r* = 0.56) but significantly negatively correlated with RTN (*r* = −0.47), RFN (*r* = −0.43), and RC (*r* = −0.48). RV maintained significant positive correlations with RTN (*r* = 0.36), RFN (*r* = 0.40), and RC (*r* = 0.26).

### 3.3. Results of Association Analysis for Rice Salt Tolerance

#### 3.3.1. GWAS Results for the Trait of Salt Tolerance Levels in Rice

**Figure 2:**
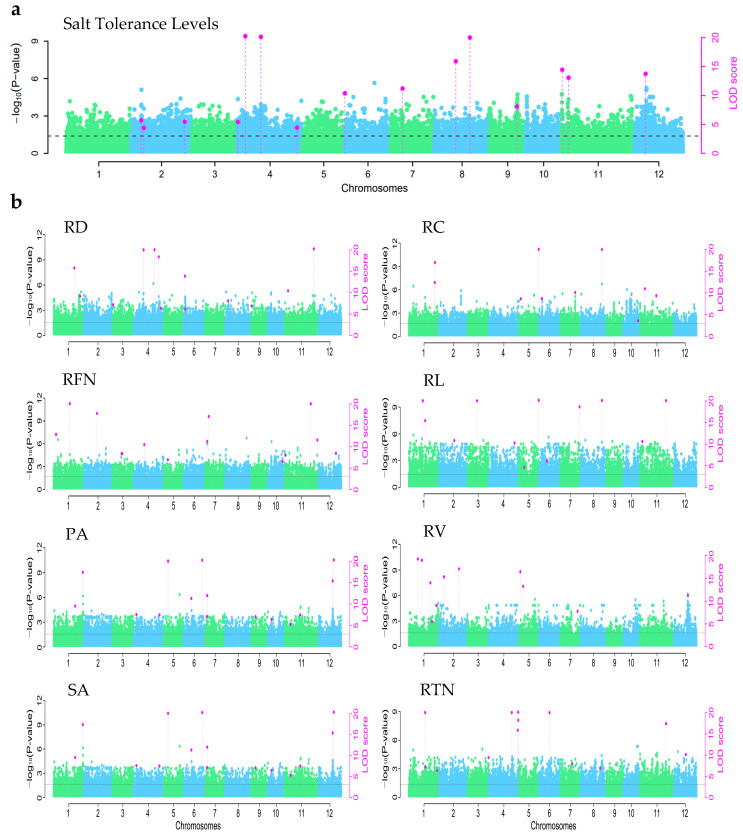
Results of salt tolerance levels and root trait localization at the rice seedling stage. Note: (**a**) Description of GWAS results for the trait of salt tolerance levels in rice; (**b**) Description of GWAS results for root morphological traits under salt stress in rice. RD, Root diameter; RC, Root crossings; RFN, Root forks number; RL, Root length; PA, Project area; RV, Root volume; SA, Surface area; RTN, Root tips number.

A total of 165 rice seedlings were analyzed by the 3VmrMLM method for GWAS based on SNP, and 15 loci were identified on 8 chromosomes (Figure 2a, Table 2). One QTN was mapped on chromosomes 5, 7, 9, and 12, explaining 5.63%, 3.51%, 4.82% and 2.58% of the phenotypic variation, respectively. Two QTNs were mapped on chromosomes 8 and 11, explaining 8.40% and 7.18%, 3.35% and 2.10% of the phenotypic variation, respectively. Three QTNs were mapped on chromosome 2, explaining 3.00%, 4 QTNs were mapped on chromosome 4, accounting for 3.19%, 5.59%, 6.69% and 1.82%, respectively.

#### 3.3.2. GWAS Results for Root Morphological Traits Under Salt Stress in Rice

A total of 78 QTNs were found to be associated with eight root morphological traits (Figure 2b, Supplementary Table S3). Among these, eight QTNs exhibited pleiotropic effects, each being associated with at least two traits. Specifically, QTN SNP1-23075257 was associated with RFN and RTN; SNP1-30068136 and SNP12-363719301 were associated with PA, RA, and SV; SNP5-180717660 and SNP8-264965843 were associated with RC and RL; SNP7-216175647 was associated with RFN, PA, and SA; SNP10-312864113 was associated with RC and RFN; and SNP11-341037053 was associated with RFN, RL, and RTN.

For RD, 13 QTNs were identified on 7 chromosomes. One QTN, named as SNP1-28814363, located on chromosome 1, was identified as accounting for the greatest proportion of phenotypic variation estimation (PVE) of 11.6%. The QTN SNP4-146715486, located on chromosome 4, explaining 6.36% phenotypic variation, was detected within the same genomic region as the previously cloned gene *OsHKT1;1* [26,27], which is known to reduce sodium accumulation in roots by regulating sodium excretion. The other three QTNs located on chromosome 4 explained the phenotypic variation of 6.15%, 3.67%, 6.36% and 2.37%, respectively. One QTN was mapped on each of chromosomes 3, 8, and 9, which explained the phenotypic variation of 4.13%, 4.12% and 4.22%. Four QTNs were located on chromosomes 6 and 11, which explained the phenotypic variation of 6.44% and 3.89%, 8.48% and 4.45%, respectively. Ten QTNs were identified to be associated with RC. Among these, SNP11-323749245 explained the greatest proportion of PVE at 8.61%, and SNP1-37702790 was colocalized with the gene locus of *OsMGT1* [28]. The other eight QTNs were located on chromosomes 1, 5, 6, 7, 8, and 11, with PVE ranging from 1.23% to 8.61%. For RFN, a total of 14 loci were identified on 9 chromosomes, among which five QTNs were located on chromosomes 2, 4, 5, 10, and 12, which explained the PVE ranging from 2.91% to 5.32%. Two QTNs were located on chromosomes 1, 3, and 7, which explained the phenotypic variation of 6.01% and 2.65%, 5.21% and 2.58%, and 1.66% and 1.87%, respectively. There were three QTNs located on chromosome 11, which explained the phenotypic variation of 4.19%, 2.41% and 4.20%, respectively. Twelve loci were identified to be related to RL on nine chromosomes, among which six QTNs were located on each of chromosomes 2, 3, 4, 6, 7, and 8, which explained the phenotypic variation of 7.33%, 2.07%, 4.83%, 3.75%, 10.56% and 6.69%, respectively. The QTN SNP7-240172795, SNP7-240172795, and SNP11-341037053 colocalize with the three salt-resistance-related genes, *OsHKT1;1* [26,27], *OsSOD* [29,30], and *OsNAC5* [31], in the same genomic regions. The other six QTNs were located on chromosomes 1, 5, and 11, which explained the phenotypic variation ranging from 2.01% to 6.29%.

Due to a perfect correlation (r = 1) between PA and SA, their GWAS results were identical. A total of 15 loci were identified on 9 chromosomes, among which three QTNs were located on chromosomes 5, 9, and 10, explaining the phenotypic variation of 3.17%, 3.25% and 3.84%, respectively. The QTN SNP4-147056819 overlapped with the previous coloned gene *OsHKT1;4* [32,33], accounting for 1.8% PVE. The rest 11 QTNs were located on chromosomes 1, 4, 6, 7, 11, and 12, which explained the PVE ranging from 1.58% to 6.66%. Eleven QTNs were identified on five chromosomes for RV. Among these, the QTN SNP1-40894850 was located in the same genomic region within the gene *OsHAK5* [34,35], which protects root cell integrity by enhancing the ability to remove reactive oxygen species. Two QTNs were, respectively, located on chromosomes 7 and 12, which explained 5.31% and 8.32% phenotypic variation. Four QTNs were located on chromosomes 2 and 5, which explained the phenotypic variation of 0.97% and 3.73%, 7.27% and 4.50%, respectively. Five QTNs were located on chromosome 1, which explained the phenotypic variation ranging from 2.52% to 7.98%. A total of 12 QTNs for RTN were detected on 8 chromosomes. Six QTNs were separately located on chromosomes 3, 6, 7, 8, 11, and 12, explaining the phenotypic variation of 6.73%, 2.90%, 5.51%, 4.15%, 2.31% and 4.53%, respectively. The detected QTN SNP1-41088412 and SNP4-149145662 coincided with the reported gene *OsHAK5* [34,35] and *OsRMC* [36,37], respectively. Three QTNs were located on chromosome 1, which explained the phenotypic variation of 3.23%, 4.04% and 4.52%, respectively. Four QTNs were located on chromosome 4, which explained the phenotypic variation ranging from 1.83% to 5.09%.

### 3.4. The Performance of the Selected Salt-Tolerance Varieties Compared to the Control Lines

**Figure 3:**
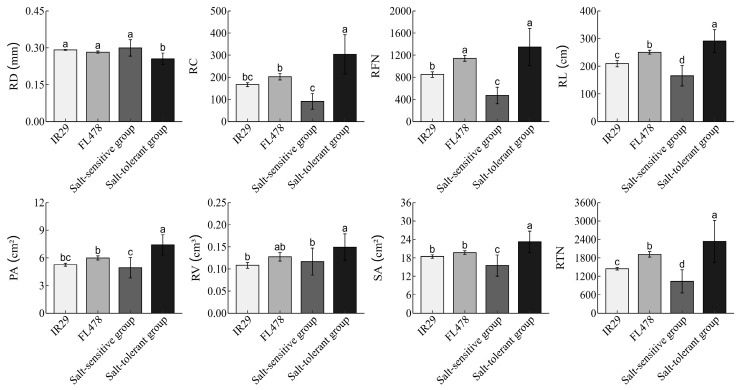
Comparison results of root traits among salt-tolerant materials, salt-sensitive materials, and the control group. Note: Different lowercase letters (a, b, c, d) indicate significant differences among means at the 0.05 probability level according to multiple comparison analysis. RD, Root diameter; RC, Root crossings; RFN, Root forks number; RL, Root length; PA, Project area; RV, Root volume; SA, Surface area; RTN, Root tips number.

Through salt tolerance evaluation at the seedling stage, a total of six salt-tolerance rice varieties were screened, including IRIS_313-8735, B017, B162, CX74, CX113, and IRIS_313-9813 (Supplementary Table S4). The overall performance of the selected salt-tolerant lines was better than that of both the salt-sensitive control line IR29 and the salt-tolerant control line FL478. Compared to the salt-sensitive control IR29, the selected salt-tolerant rice lines showed significant improvements in eight root morphological traits. The salt-tolerance group exhibited a 0.04 mm (12.51%) decrease in RD, an 81.91 cm (39.14%) increase in RL, a 136.42 (81.81%) increase in RC, a 497.83 (58.60%) increase in RFN, an 81.91 cm (39.14%) increase in RL, a 2.14 cm^2^ (40.69%) increase in PA, a 0.04 cm^3^ (37.93%) increase in RV, a 4.82 cm^2^ (26.14%) increase in SA, and an 889.08 (61.50%) increase in RTN. The salt-sensitive group was significantly lower than the salt-sensitive control line IR29 in RFN, RL, SA, and RTN, but there were no significant differences in the other four traits (Figure 3).

Compared to the salt-tolerance variety FL478, the selected salt-tolerance group showed notable improvements in RD, RC, RFN, RL, PA, RV, SA, and RTN. Specifically, the salt-tolerant group experienced an increase of 100.67 (49.71%) in RC, 204.83 (17.93%) in RFN, 40.45 cm (16.13%) in RL, 1.42 cm^2^ (23.66%) in PA, 0.02 cm^3^ (16.99%) in RV, 3.52 cm^2^ (17.83%) in SA, and 420.33 (21.96%) in RTN. In contrast, there was a significant decrease of 0.03 mm (9.74%) in RD (Figure 3).

### 3.5. Analysis of Favorable Alleles for the Selected Salt-Tolerance Lines

**Figure 4:**
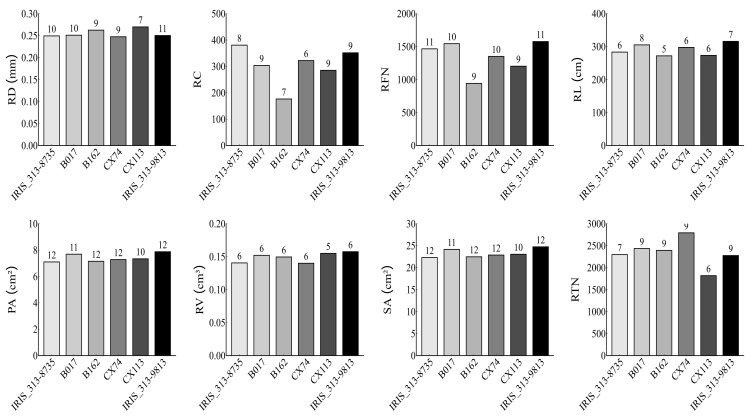
The performance of root morphological traits of salt-tolerance lines with different favorable alleles. Note: The numbers labeled above the bars represent the counts of corresponding favorable alleles. RD, Root diameter; RC, Root crossings; RFN, Root forks number; RL, Root length; PA, Project area; RV, Root volume; SA, Surface area; RTN, Root tips number.

We further analyzed the distribution of favorable alleles across the six salt-tolerance lines (Figure 4). For the 13 QTNs linked to RD, the six salt-tolerant varieties contained 10, 10, 9, 9, 7, and 11 favorable alleles, respectively. Although both B162 and CX74 carried 9 favorable alleles, CX74 exhibited a more favorable phenotypic value, likely due to the presence of the large effective allele SNP1-28814363. Regarding the 10 QTNs associated with RC, the six lines carried 8, 9, 7, 6, 9, and 9 favorable alleles, respectively. For RFN, 14 QTNs were identified. IRIS_313-8735 carried 11 favorable alleles, and the remaining five lines contained 10, 9, 10, 9, and 11 alleles, respectively. Among the 12 QTNs related to RL, IRIS_313-8735 possessed 6 favorable alleles, while the other lines carried 8, 5, 6, 6, and 7, respectively. For the 15 related to PA, the six salt-tolerance varieties contained 12, 11, 12, 12, 10, and 12 favorable alleles, respectively. For RV, 11 QTNs were detected, with each line carrying 6, 6, 6, 6, 5, and 6 alleles, respectively. Similarly, 15 QTNs were identified for SA, distributed as 12, 11, 12, 12, 10, and 12 across the lines. For RTN, 13 QTNs were associated with RTN, with the lines carrying 7, 8, 9, 8, 8, and 9 alleles, respectively. The results demonstrated a positive correlation between the number of favorable alleles and improved performance.

## 4. Discussion

A complex causal relationship exists between the root system of rice and salt tolerance at the seedling stage. Multiple research studies have conducted extensive investigations into the association between rice root systems and salt tolerance. Zang (2008) used a BC_2_F_8_ backcross introgression line population derived from a cross between moderately salt-sensitive *indica* rice IR64 and *japonica* rice Tarom molaii to detect 23 QTLs associated with salt tolerance at the seedling stage [38]. The study conducted by Li (2018) focused on functional characterization of a specific gene, demonstrating that overexpression of RCc3 significantly improves root system architecture—such as by increasing root length and biomass—and enhances salt tolerance at the seedling stage, indicating that genetic modulation of root development represents an effective strategy for improving salinity stress resistance [39]. In contrast, Xu et al. (2024) employed a population genetics approach, integrating genome-wide association studies (GWAS) and linkage analysis to systematically identify major quantitative trait loci (QTLs) controlling salt tolerance in *japonica* rice seedlings [40]. Among these, several loci were implicated in the regulation of root morphological traits, providing a valuable repository of candidate genes for breeding salt-tolerant rice varieties. Collectively, these findings underscore that optimization of root system architecture serves as an essential foundation for enhancing salt tolerance in rice. Therefore, the root system plays a crucial role in salt tolerance at the seedling stage in rice.

Compared with previous studies, our study innovatively utilized the ScanMaker i850 plus root scanner to systematically measure eight root morphological traits, such as average root diameter, root crossings number, root forks number, root length, root projection area, root volume, root surface area, and root tip number. A total of 78 QTNs related to the eight root morphological traits were identified, among which 7 were co-localized with known salt-tolerance genes. The identified QTN, named as SNP4-146715486, was found to be located within the same genomic region as the previously cloned gene *OsHKT1;1* [26,27], which is known to reduce sodium accumulation in roots by regulating sodium excretion. The SNP1-40894850 was detected to localize within the same genomic region as *OsHAK5* [34,35], and the SNP7-240172795 was identified to reside in the same genomic region as *OsSOD* [29,30]; both of these loci protect root cell integrity by enhancing ROS scavenging ability. This achievement greatly enriches our understanding of the genetic mechanism of rice root salt tolerance and provides more comprehensive and precise theoretical support and gene targets for the subsequent selection of salt-tolerant rice varieties.

Under salt stress, genetic regulation and physiological adaptations, such as the maintenance of ion homeostasis and enhanced antioxidant defense, together shape the root system architecture of salt-tolerant rice; these adaptive changes further converge to form a more efficient nutrient uptake network. Building on this understanding of root architectural plasticity under salt stress, its profound biological significance is primarily manifested in the regulation of core physiological mechanisms and the establishment of foundational conditions for seedling growth. In our study, the selected salt-tolerant accessions also demonstrated significant improvements across multiple morphological traits. The salt-tolerant group exhibited an increase in RL, PA, SA, RTN, and RFN. These enhanced traits provide more functional sites for transporters, which not only strengthen the “Na^+^-extrusion-K^+^ retention” effect and selective ion uptake but also enable precise regulation of ion influx and efflux by modulating the activity of ion channels [41]. Meanwhile, the elongated roots can penetrate the surface high-salt zone and block apoplastic Na^+^ transport via the casparian strip. For antioxidant defense, it not only increases the distribution sites of enzymes such as superoxide dismutase (SOD) and peroxidase (POD) to scavenge reactive oxygen species (ROS) but also regulates the metabolism of antioxidant substances through ROS/calcium signaling pathways [42]. Correlation analysis revealed strong positive associations among these root traits, indicating that salt-tolerant lines enhance nutrient acquisition efficiency through increased root length and greater branching complexity. These results suggest that salt-tolerant rice varieties effectively adapt to saline environments by optimizing root architecture, including increased root elongation, expanded surface area, and enhanced lateral branching, which collectively support sustained nutrient uptake and maintained shoot growth under salt stress.

Compared to the salt-tolerant control variety FL478, the selected salt-tolerant lines carried more favorable alleles than the salt-sensitive lines. For instance, the line IRIS_313-8735 harbored ten favorable alleles associated with root diameter (RD), which were predominantly enriched in biological pathways related to root development and ion transport. These genetic features contribute to improved water uptake efficiency, regulation of aquaporin function, and increased sites for the synthesis and accumulation of osmolytes, thereby helping maintain cellular water balance and osmotic stability [43,44]. Moreover, the six selected salt-tolerant lines carrying the favorable allele of locus SNP4-147056819 showed significantly increased PA and SA, further supporting the genetic relationship between root morphology and salt tolerance. There are three pathways, including ion exclusion, osmotic adjustment, and antioxidant defense, that act synergistically to significantly improve the salt tolerance of rice at the seedling stage, while ensuring the normal growth of shoots, a pivotal hub for nutrient translocation. This lays an early physiological foundation for the establishment of “source-sink-flow” balance in subsequent growth stages of rice, thereby sustaining the overall growth potential of the plant.

Based on the content discussed above, root scanning phenotypes can serve as key indicators for evaluating salt tolerance at the seedling stage, thereby providing a robust basis for screening and breeding salt-tolerant rice materials. Meanwhile, Future studies should incorporate dynamic root phenotyping across multiple developmental stages to elucidate the functional links between root system architecture and yield formation under saline conditions.

## Figures and Tables

**Table 1:** Descriptive statistics of root-related traits.

	Root Diameter (RD) (mm)	Root Crossings (RC)	Root Forks Number (RFN)	Root Length (RL) (cm)	Project Area (PA) (cm^2^)	Root Volume (RV) (cm^3^)	Surface Area (SA) (cm^2^)	Root Tips Number (RTN)
Mean	0.29	154.65	795.59	211.80	6.09	0.14	19.14	1440.17
Standard deviation	0.03	72.22	279.92	52.21	1.42	0.04	4.45	469.82
Skewness	0.67	0.98	0.93	0.80	0.63	0.78	0.63	0.49
Kurtosis	0.67	0.75	1.07	1.51	0.96	0.61	0.96	0.06
CV (%)	10.34	46.70	35.18	24.65	23.32	28.57	23.25	32.62
Minimum	0.21	30.50	238.50	109.54	3.34	0.07	10.50	346.50
Maximum	0.41	389.50	1819.50	439.94	11.64	0.29	36.56	2792.00

**Table 2:** Translation of salt tolerance levels localization results at the rice seedling stage.

Marker	Chromosome	Position (bp)	LOD	PVE (%)	Favorable Allele
SNP2-49524748	2	6,253,825	5.71	3.00	C
SNP2-51399642	2	8,128,719	4.40	2.38	T
SNP2-74166363	2	30,895,440	5.46	1.42	G
SNP4-115698850	4	76,858	5.41	3.19	G
SNP4-117616752	4	1,994,760	45.08	5.59	A
SNP4-123092399	4	7,470,407	32.88	6.69	T
SNP4-147472833	4	31,850,841	4.44	1.82	C
SNP5-180922039	5	297,97,353	10.37	5.63	A
SNP7-221576010	7	9,244,103	11.20	3.51	T
SNP8-252176489	8	10,146,961	15.91	8.40	A
SNP8-260086690	8	18,057,162	20.55	7.18	G
SNP9-286257626	9	15,785,076	8.11	4.82	C
SNP11-317386697	11	694,140	14.44	3.35	G
SNP11-322275422	11	5,582,865	13.07	2.10	G
SNP12-354603209	12	8,889,546	13.74	2.58	A

## Data Availability

The original contributions presented in the study are included in the article/Supplementary Material. Further inquiries can be directed to the corresponding author. MDPI Research Data Policies.

## Supplementary Materials

The following supporting information can be downloaded at: https://www.mdpi.com/article/10.3390/life15101595/s1, Table S1: Yoshida nutrient solution formula; Table S2: Standard evaluation score of visual salt injury at the seedling stage; Table S3: Translation of root trait localization results under salt stress at rice seedling stage using agricultural terminology; Table S4: Rice germplasm resources with strong salt tolerance and salt sensitivity screened based on salt damage level at seedling stage.

## Abbreviations

The following abbreviations are used in this manuscript:

RD	Root diameter
RC	Root crossings
RFN	Root forks number
RL	Root length
PA	Project area
RV	Root volume
SA	Surface area
RTN	Root tips number
