# Supplementary material for: Unveiling Genetic Loci for Root Morphology and Salt Response at Rice Seedling Stage via Genome-Wide Association Studies
Source: Life (Basel). 2025 Oct 13;15(10):1595. doi: 10.3390/life15101595 (PMC12565415; doi:10.3390/life15101595)
Supplement: Supplementary file 1 [file life-15-01595-s001.zip › life-3876230-supplementary.pdf]

Supplementary Table S1. YSoshida nutrient solution formula.

Component	Reagent Name (Analytical Grade)	Grams/4 L stock solution	Stock Solution Volume (mL)	final concentration (mg/L)
Macronutrients				
N	$(NH_4)_2SO_4$	603.2		40
P	$NaH_2PO_4 \cdot 2H_2O$	160.9		10
K	K_2SO_4	285.6	450	40
Ca	$CaCl_2 \cdot 2H_2O$	469.4		40
Mg	$MgSO_4 \cdot 7H_2O$	1296.0		40
Micronutrients				
Mn	$MnCl_2 \cdot 4H_2O$	6.000		0.50
Mo	$(NH_4)_6Mo_7O_{24} \cdot 4H_2O$	0.296		0.05
Zn	$ZnSO_4 \cdot 7H_2O$	0.140		0.01
B	H_3BO_3	3.736	450	0.20
Cu	$CuSO_4 \cdot 5H_2O$	0.124		0.01
Fe	$FeCl_3 \cdot 6H_2O$	30.800		2.00
Citric Acid	$C_6H_8O_7 \cdot H_2O$	47.600		

Supplementary Table S2.

Standard evaluation score of visual salt injury at the seedling stage.

Grade	Symptoms	Salt-Tolerance Grade
1	The growth is normal and the leaves show no symptoms	Highly resistant
3	Nearly normal growth, with white or curled leaf tips	Resistant
5	Growth is significantly inhibited, with most leaves curing up and only a few leaves straightening out	Moderately resistant
7	Growth stops, most leaves dry up, and some plants die	Moderately susceptible
9	Almost all the plants died or were close to death	Susceptible

Supplementary Table S3. Translation of root trait localization results under salt stress at rice seedling stage using agricultural terminology.

Trait	Marker	Chromosome	Position (bp)	LOD	PVE(%)	Favorable Allele	Related gene
	SNP1-28814363	1	28814363	15.75	11.60	G	
	SNP1-38711626	1	38711626	9.15	3.85	T	
	SNP3-81243817	3	2035644	7.17	4.13	T	
	SNP4-122086285	4	6464293	21.18	6.15	C	
	SNP4-137917787	4	22295795	21.45	3.67	C	
	SNP4-146715486	4	31093494	18.37	6.36	A	*OsHKT1;1*[27,28]
RD	SNP4-147404431	4	31782439	6.32	2.37	C	
	SNP6-182609733	6	1526613	13.84	6.44	C	
	SNP6-182776671	6	1693551	6.18	3.89	A	
	SNP8-245179195	8	3149667	8.06	4.12	C	
	SNP9-271340655	9	868105	6.86	4.22	G	
	SNP11-322383973	11	5691416	10.43	8.48	C	
	SNP11-342752611	11	26060054	41.61	4.45	C	
	SNP1-37509113	1	37509113	12.41	1.26	C	*OsMGT1*[28]
	SNP1-37702790	1	37702790	17.01	4.65	G	*OsMGT1*[28]
	SNP5-152612264	5	1487578	8.65	1.23	T	
	SNP5-180717660	5	29592974	23.40	4.59	T	
RC	SNP6-185238749	6	4155629	8.72	7.95	A	
	SNP7-233802105	7	21470198	10.10	6.40	A	
	SNP8-264965843	8	22936315	21.70	5.36	A	
	SNP10-312864113	10	19378843	3.61	2.46	G	
	SNP11-323749245	11	7056688	10.95	8.61	C	
	SNP11-335482580	11	18790023	9.37	4.28	G	
	SNP1-3447129	1	3447129	12.87	6.01	T	
	SNP1-23075257	1	23075257	23.07	2.65	A	
	SNP2-63635110	2	20364187	17.78	2.95	G	
	SNP3-96185899	3	16977726	8.34	5.21	A	
	SNP3-7089415	3	17881242	8.40	2.58	T	
	SNP4-122907821	4	7285829	10.48	4.42	T	
	SNP5-156082010	5	4957324	6.98	2.91	G	
RFN	SNP7-216175647	7	3843740	11.22	1.66	G	
	SNP7-219521644	7	7189737	17.06	1.87	C	
	SNP10-312864113	10	19378843	6.54	3.28	G	
	SNP11-318921721	11	2229164	8.06	4.19	C	
	SNP11-341037053	11	24344496	20.89	2.41	C	
	SNP11-344507042	11	27814485	11.57	4.20	T	
	SNP12-366994689	12	21281026	8.44	5.32	T	
RL	SNP1-20202744	1	20202744	23.29	2.01	T	

	SNP1-23118199	1	23118199	15.43	2.71	C	
	SNP2-66495205	2	23224282	10.84	7.33	C	
	SNP3-96394440	3	17186267	22.00	2.07	T	
	SNP4-146985464	4	31363472	10.25	4.83	G	*OsHKT1;1*[26,27]
	SNP5-157649529	5	6524843	4.60	2.26	C	
	SNP5-180717660	5	29592974	34.65	6.29	T	
	SNP6-191784508	6	10701388	6.11	3.75	T	
	SNP7-240172795	7	27840888	18.62	10.56	A	*OsSOD*[29,30]
	SNP8-264965843	8	22936315	30.74	6.69	A	
	SNP11-321170053	11	4477496	10.61	3.49	G	*OsNAC5*[31]
	SNP11-341037053	11	24344496	26.57	3.66	C	
	SNP1-30068136	1	30068136	9.53	3.77	C	
	SNP1-42082956	1	42082956	17.38	2.79	C	
	SNP4-116978736	4	1356744	7.54	3.77	C	
	SNP4-147056819	4	31434827	7.44	1.80	T	*OsHKT1;4*[32,33]
	SNP5-156467382	5	5342696	23.20	3.17	C	
	SNP6-191555975	6	10472855	11.32	2.79	C	
PA	SNP6-208170213	6	27087093	44.48	6.66	C	
	SNP7-215613083	7	3281176	7.07	3.62	C	
	SNP7-216175647	7	3843740	11.99	1.58	G	
	SNP9-277217516	9	6744966	7.05	3.25	A	
	SNP10-297089701	10	3604431	6.43	3.84	C	
	SNP11-324700078	11	8007521	5.33	2.66	A	
	SNP11-334941141	11	18248584	7.48	4.30	C	
	SNP12-363719301	12	18005638	15.39	7.89	G	
	SNP12-364422141	12	18708478	51.10	4.31	G	
	SNP1-13816163	1	13816163	19.29	6.00	G	
	SNP1-18971694	1	18971694	19.00	2.52	C	
	SNP1-30068136	1	30068136	14.04	5.61	C	
	SNP1-32272545	1	32272545	5.50	5.69	T	
	SNP1-40894850	1	40894850	9.02	7.98	A	*OsHAK5*[34,35]
RV	SNP2-50025126	2	6754203	15.37	0.97	T	
	SNP2-68087640	2	24816717	17.10	3.73	C	
	SNP5-152144024	5	1019338	16.49	7.27	A	
	SNP5-155953349	5	4828663	13.28	4.50	T	
	SNP7-236886239	7	24554332	7.77	5.31	A	
	SNP12-363719301	12	18005638	11.23	8.32	G	
	SNP1-30068136	1	30068136	9.53	3.77	C	
	SNP1-42082956	1	42082956	17.38	2.79	C	
SA	SNP4-116978736	4	1356744	7.54	3.77	C	
	SNP4-147056819	4	31434827	7.44	1.80	T	*OsHKT1;4*[32,33]

	SNP5-156467382	5	5342696	23.20	3.17	C	
	SNP6-191555975	6	10472855	11.32	2.79	C	
	SNP6-208170213	6	27087093	44.48	6.66	C	
	SNP7-215613083	7	3281176	7.07	3.62	C	
	SNP7-216175647	7	3843740	11.99	1.58	G	
	SNP9-277217516	9	6744966	7.05	3.25	A	
	SNP10-297089701	10	3604431	6.43	3.84	C	
	SNP11-324700078	11	8007521	5.33	2.66	A	
	SNP11-334941141	11	18248584	7.48	4.30	C	
	SNP12-363719301	12	18005638	15.39	7.89	G	
	SNP12-364422141	12	18708478	51.10	4.31	G	
RTN	SNP1-23075257	1	23075257	24.11	3.23	A	
	SNP1-23271312	1	23271312	7.12	4.04	C	
	SNP1-41088412	1	41088412	6.30	4.52	T	*OsHAK5*[34,35]
	SNP3-115218226	3	36010053	9.41	6.73	T	
	SNP4-141932133	4	26310141	22.89	2.17	T	
	SNP4-148385158	4	32763166	15.87	1.83	T	
	SNP4-149145662	4	33523670	31.07	5.09	T	*OsRMC*[36,37]
	SNP6-195171616	6	14088496	20.60	2.90	G	
	SNP7-231201444	7	18869537	7.89	5.51	A	
	SNP8-262192369	8	20162841	6.90	4.15	C	
	SNP11-341037053	11	24344496	17.42	2.31	C	
	SNP12-362416986	12	16703323	10.12	4.53	G	

Supplementary Table S4. Rice germplasm resources with strong salt tolerance and salt sensitivity screened based on salt damage level at seedling stage.

Germplasm Type	Material Name	IRIS_ID	Subgroup
Salt-tolerant germplasm	NEP NGAU::IRGC 78369-1	IRIS_313-8735	GJ-adm
	Aerjituo	B017	GJ-tmp
	Baigedao	B162	GJ-tmp
	JP-5	CX74	GJ-tmp
	Biniapan	CX113	GJ-trp
	SZANISZLO 2::IRGC 9353-1	IRIS_313-9813	GJ-tmp
